# The development and validation of a measurement instrument to investigate determinants of health care utilisation for low back pain in Ethiopia

**DOI:** 10.1371/journal.pone.0227801

**Published:** 2020-01-16

**Authors:** Getahun Kebede Beyera, Jane O’Brien, Steven Campbell

**Affiliations:** 1 School of Nursing, College of Health and Medicine, University of Tasmania, Launceston, Tasmania, Australia; 2 Institute of Public Health, College of Medicine and Health Sciences, University of Gondar, Gondar, Ethiopia; University of Lleida, SPAIN

## Abstract

**Introduction and objectives:**

Low back pain (LBP) is a highly prevalent and disabling public health problem globally. However, little is known about factors affecting health care utilisation for optimal management of the pain, and there is no validated instrument to derive epidemiological data for a better understanding of these factors. The aim of this study was to develop and validate an instrument used to measure determinants of health care utilisation for LBP in Ethiopia.

**Methods:**

The relevant domains of potential determinants of health care utilisation for LBP were identified following a comprehensive review of the literature. Items relating to each domain were then generated by considering the context of Ethiopia, and where necessary, existing items were adapted. The instrument was then translated, and an expert panel reviewed the instrument for content validity, clarity and any other suggestions. Using the data collected from 1303 adults with LBP, factorial validity was assessed by conducting principal component and parallel analyses. Internal consistency reliability was also assessed using Cronbach’s *alpha*. Intraclass correlation coefficient (ICC) and Cohen Kappa statistic were calculated to evaluate temporal stability of the instrument.

**Results:**

Parallel analysis showed that there were six components with Eigenvalues (obtained from principal component analysis) exceeding the corresponding criterion values for a randomly generated data matrix of the same size. Cronbach’s *alpha* for the internal consistency reliability ranged from 0.65 to 0.82. In assessing temporal stability, ICC ranged from 0.60, 95% CI: 0.23–0.98 to 0.95, 95% CI: 0.81–1.00 while Cohen Kappa ranged from 0.72, 95% CI: 0.49–0.94 to 0.93, 95% CI: 0.85–1.00.

**Conclusions:**

This study demonstrated that the newly developed instrument has an overall good level of content and factorial validity, internal consistency reliability, and temporal stability. In this way, this instrument is appropriate for measuring determinants of health care utilisation among people with LBP in Ethiopia.

## Introduction

Musculoskeletal disorders (MSDs) are major problems for public health systems in the world [1, 2]. The term MSDs is broad and includes a range of disorders, from those of acute onset and short duration to lifelong disorders, such as low back pain (LBP), arthritis, and osteoporosis [2, 3]. This diverse group of disorders cause a high economic and personal burden to society and the individual, including utilisation of health resources, disruptions to daily life, and loss of productivity due to functional limitations and activity restrictions [4].

LBP, which remains the most prevalent form of MSDs, is a global epidemic health problem across the course of human life [5]. It is a condition responsible for significant social impact, particularly being an important source of demand for health services [6, 7]. Over the past 25 years (1990 to 2015), Disability Adjusted Life Years (DALYs), caused by LBP alone, increased by 54% worldwide, with the greatest proportion of increase observed in low- and middle-income countries [8]. This DALYs caused by LBP is most prevalent among economically productive age groups, particularly in low- and middle-income countries, where informal employment is common, and there is no or limited possibility of job modification [9]. Evidence shows that individuals with LBP are more often diagnosed with other conditions, such as psychological disorders, musculoskeletal pain, and somatoform disorders [10]. These comorbid conditions are associated with poor outcomes, including diminishing health related quality of life [11, 12]. The rise in the prevalence and associated impact of LBP have led to an increasing array of tests and treatment utilisation, including injections, surgical procedures, implantation devices, and medications [13]. This shows that LBP strains the limited and already overburdened health care and social systems in both low- and middle-income countries [14]. Health care costs due to LBP constitute a significant burden to individuals, society and public health care systems [15]. Thus, LBP is a growing public health problem in low- and middle-income countries with far-reaching public health consequences.

A study undertaken by Misganaw et al [16] showed that LBP is one of the top ten causes of age standardised DALYs in Ethiopia. The same study further demonstrated that from 1990 to 2015, while DALYs caused by all other top 30 contributors (such as measles, malaria and protein energy malnutrition) were shown to decrease, DALYs caused by LBP and sense organ diseases continued to increase. This shows that, combined with neglected tropical diseases, HIV/AIDS, tuberculosis, malaria, and anaemia, which are the common causes of DALYs in sub-Saharan Africa [17], LBP may pose a serious burden in Ethiopia. Despite no population-based study that has investigated the epidemiology of LBP in Ethiopia, the data generated from certain segments of working population show a high prevalence of LBP [18–20]. For example, a study conducted by Beyen et al [18] indicated that the lifetime and annual prevalence of LBP mong teachers were 57.5% and 53.8%, respectively. Moreover, psychological factors, such as symptoms of depression, anxiety, and post-traumatic stress, were ascertained to be potential risk factors associated with the higher prevalence of LBP in rural Ethiopia [20]. In general, there is evidence [6] to argue that LBP, particularly in the chronic phase poses a considerable challenge to the research agenda of low-income countries, and therefore, needs to be assessed and monitored. Combined with frequent use of health services, LBP leads to long-term disability [21]. As a result, the number of studies investigating the effectiveness of interventions in LBP patients has been significantly increased over the last two decades [22]. These studies showed that the patterns of health care utilisation for LBP vary geographically [23], and are lacking in low-income countries such as Ethiopia [24]. However, the data on health care utilisation for LBP can be used for several purposes, including monitoring the health and wellbeing of the population struggling with the pain, and to develop appropriate strategies promoting evidence-based interventions [24]. Such data can also be useful for clinicians to plan and implement optimal treatment of the pain [25]. It is also important that a psychometrically sound measurement instrument is the foundation of rigorous research design [26]. The aim of this study was therefore to develop and validate a measurement instrument to investigate determinants of health care utilisation for LBP in Ethiopia.

## Methods

The instrument was developed according to the following multi-step process:

1. Literature review and identification of key components/domains;
2. Development of a draft measurement instrument;
3. Review by the research team and refining the instrument;
4. Forward and backward translation of the instrument (English ⇄ Oromo language);
5. Review by an expert panel and content validity determination; and
6. Data collection and evaluation of psychometric properties of the instrument.

Each of these key steps are discussed in the following sections.

### Literature review and identification of key components/domains

In order to identify potential factors influencing health care utilisation for LBP, a comprehensive review of current literature [24, 25, 27], theories [28, 29], and models of health services utilisation [30, 31] was conducted. Various studies investigating potential factors influencing health utilisation for LBP have documented different factors and categorised them under different domains. For example, Woodhouse et al [25] categorised the different factors that influence health care utilisation for LBP as: *sociodemographic factors*, such as age, gender, and marital status; *pain related factors*, such as pain intensity and work limitation due to LBP; and *other health related factors*, such as self-reported general heath, somatic health including presence of other musculoskeletal pain, other medical conditions, and mental distress such as anxiety, depression, and insomnia. Using the Andersen’s behavioural model of health services utilisation [30], Tiira et al [27], alternatively, classified factors affecting health care utilisation for LBP into four domains. These include *enabling resources*, which include socio-economic position, employment status, and residential place; *need factors*, such as intensity of pain, duration of pain, and limitations to daily activities caused by LBP; *personal health habits*, such as physical activity level, sitting time, smoking and obesity level; and *psychological factors*, including anxiety and distress. A systematic review and meta-analysis of the literature [24] also documented the factors in a manner Woodhouse et al [25].

There is no specific theory or model, to investigate the determinants of health care utilisation for LBP. Each theory and model of health services utilisation, however, make justifications differently. For example, the health belief model [31] emphasises each individual’s perception, attitude, and belief, while the social cognitive theory [28, 29] pays attention to individual, behavioural, and environmental factors. However, unlike the health belief model and the social cognitive theory approaches, most health problems, including LBP, as being complex, caused by multiple factors such as personal, socio-cultural, and environmental factors, which in turn may also influence health care utilisation behaviours of individuals involved [30, 32].

Thus, based the context of contemporary literature, theories, and models, other than sociodemographic factors, the most commonly identified determinants of health care utilisation for LBP included: (1) health behaviour/lifestyle habits, such as smoking, alcohol consumption, khat (a plant with leaves and stem tips which are chewed for their stimulating effect) chewing, body mass index (BMI) and exercise; (2) beliefs about LBP; (3) pain related factors, including nature and duration of the pain, pain interference with social and daily activities, and intensity of the pain; (4) general health related factors, such as general health status, comorbidity, depressive symptoms and sleeping problem/insomnia; and (5) factors related to accessibility to health services, such as transportation systems and income levels.

### Development of a draft measurement instrument

Once the key components/domains were identified, relevant scales and questions relating to each domain were then developed by considering the context of Ethiopia, and where necessary, existing items were adapted (the source authors of the questions were contacted, and permissions were sought to use and/or adapt the previously developed measures [33, 34]). Accordingly, 43 items assessing the five domains, 13 sociodemographic questions and 12 items assessing health care utilisation for LBP, totalling 68 items were initially developed. The research team then reviewed the items individually and reduced the total number of items to 67. The team also assessed clarity of the instrument. Discussion on each question with eight people of non-medical background was also made to further ensure clarity and face validity of the instrument.

### Translation (English ⇄ Oromo language)

The translation of the instrument in this study was carried out in accordance with a guideline for instrument development and/or translation [35] and perspectives in the literature [36, 37]. The translation procedure had three stages, forward and backward translation, followed by an expert committee discussion to produce the final version of the translation. Two individuals participated in the forward translation. The guideline [35] demonstrates that in order to provide a reliable equivalency, one of the translators must be aware of the constructs the instrument intended to measure. The second translator should neither be aware, nor informed, of the concepts in order to offer a translation that produces the language used by the general public. Accordingly, one of the investigators (GKB) and other person (with no medical background) who did not know the purpose of the instrument completed the translation independently. In accordance with the literature [35, 36], both translators then discussed and resolved inconsistencies until a synthesis of the translation was reached. To avoid bias, it is recommended that the backward translators should be blind to the original version of the instrument [35, 37]. Two bilingual individuals, who did not know the intended concepts of the instrument, then translated the Oromo language version of the instrument backward to English independently. Differences were then discussed and resolved later. The final step is for the version of the translation to be agreed upon by an expert committee [35]. The members of this expert committee are suggested to be composed of both the forward and backward translators, individuals who developed the original instrument, and experts well familiar with the constructs that the instrument is intended to measure. Accordingly, all individuals participated in the forward and backward translation, and one of the developers of the original instrument (GKB), together reviewed each item in all versions of the instrument. In producing the final version of the translation, inconsistencies were resolved through discussion.

### Review by an expert panel and content validity assessment

Content validity refers to the extent to which a measurement instrument has an appropriate sample of items to represent the concept to be measured [26, 38, 39]. Content validity assessment is the initial step in establishing the validity of a measurement instrument. This largely relies upon experts’ judgement of the instrument, and thus, the soundness of the content validity assessment is significantly influenced by how the experts chosen and utilised to develop the instrument [40]. Three public health experts working in Regional State Health Bureau, four specialists in neurology working in specialised hospital, two public health researchers working in higher education, and one health services consultant working in a private organisation for holistic health service and management consultancy, totalling 10 senior experts were invited to review and evaluate the content of the instrument. As per the literature [41, 42], the selection of experts was based on their research experiences in health services and/or in management of chronic diseases such as LBP and other spinal pain. The experts were then requested to evaluate the relevance of the instrument on a four point ordinal scale (1 = not relevant, 2 = somewhat relevant, 3 = quite relevant, and 4 = highly relevant) as recommended by Polit et al [39], and to suggest any other items that might be omitted from the instrument. In order to compute the content validity index of each item (I-CVI), the ordinal rating scale was dichotomised as relevant (ratings of 3 and 4) and not relevant (ratings of 1 and 2). I-CVI for each item was then calculated as the number of experts rating relevant divided by the total number of experts. When the number of experts rating the instrument is five or fewer, the I-CVI should not be less than one for the item to be content valid, meaning that all the experts must rate the item as content valid [39]. Alternatively, when the number of experts is more than five, it is possible to accept a modest amount disagreement among the experts.

To adjust for the chance agreement among the experts participated in rating the content validity of the instrument, modified Kappa statistic (K*) was further computed using the formula, $K^{*}=\frac{\left(I-CVI-pc\right)}{\left(1-pc\right)}$. The probability of chance agreement among the experts (pc), was computed using the formula for binomial random variable, $pc=[\frac{N!}{A!(N-A)!}]0.5^{N}$. Where, N- is the number of experts and A- is the number of experts agreeing on the relevance of an item. Finally, four items (three from sociodemographic domain and one item from the domain of accessibility to health services) with I-CVI and K* less than 0.80 and 0.79, respectively were removed, resulting into 63 items. The experts’ feedback also included suggestions related to formatting and wording of the questions, which were revised accordingly. A revised version of the instrument, containing 63 items was the final product.

### Data collection and evaluation of psychometric properties of the instrument

To assess reliability and validity of the instrument, data were collected from a random sample of 1303 adults (18 years or older) with LBP residing in the general population of South-West Shewa zone of Oromia regional state. In terms of availability of health care resources and patterns of health care utilisation for LBP, South-West Shewa zone is typical of the other parts of the country. However, as the zone shares border with other regional states of the country, such as Southern Nations, Nationalities, and People’s Region (SNNPR), relatively, a diverse group of population with different socio-cultural backgrounds reside in the area. For this reason, South-West Shewa zone was chosen as a pilot site to enhance applicability of the findings of the study at the country level. The participants were selected using a multistage sampling method. Firstly, one urban centre and two rural districts within the zone were selected using OpenEpi Random number generator [43]. By the same method, two kebeles (wards) were then drawn from each of the three districts, giving a total of six kebeles. Proportional systematic random sampling was then applied to select households within the selected kebeles. Finally, one adult with LBP was selected from each household and the data were collected using face-to-face interview technique which took into account the low literacy level of the study participants.

Exploratory factor analysis (EFA) was carried out to determine factorial validity of the instrument. Prior to extraction of factors, Kaiser-Meyer-Olkin (KMO) measure of sampling adequacy was checked to evaluate the fitness of the data for factor analysis. Initially, Principal component analysis (PCA) with Varimax rotation was used to extract factors based on multiple criteria, including Eigenvalue >1, the Scree test, factor loading coefficient >0.4 and the cumulative percent of variance extracted. Finally, parallel analysis was carried out to determine the ultimate factors to be retained.

**Fig 1:**
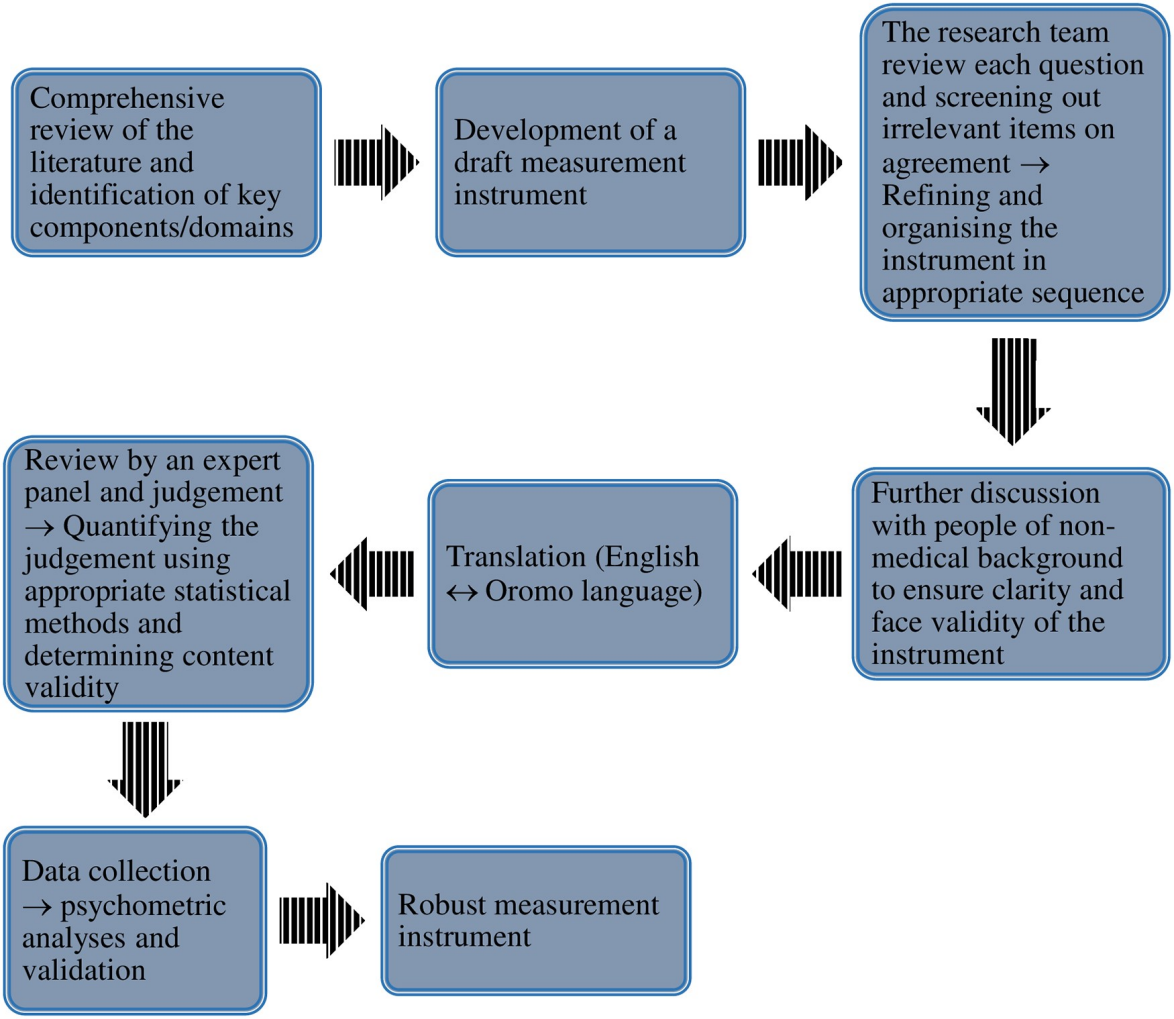
Summary of the key steps followed to develop and validate the instrument.

The internal consistency reliability of each factorially derived scale was assessed by calculating Cronbach’s *alpha*. Test-retest reliability (also called reproducibility or temporal stability) of the instrument was examined over a period of one-month, using 37 people with chronic LBP. The one-month time lapse between test and retest was intended to decrease participants’ recall bias. The intraclass correlation coefficient (ICC) was then calculated to assess the test-retest reliability of the instrument. There is evidence that “rather than a simple percent agreement, Kappa takes into account the agreement that could be expected by chance alone” [44]. For this reason, Cohen Kappa coefficient was computed to examine the test-retest reliability further. It has been argued in the literature [45, 46] that ICC/Kappa value of ≥0.70 is acceptable. PCA and Cronbach’s *alpha* calculation were carried out in Statistical Package for Social Sciences (SPSS) version 23.0 whilst ICC and Cohen Kappa were computed using R version 3.5.1. Fig 1 summarises the steps followed to develop the instrument.

### Ethical clearance

Ethical approval was obtained from the Human Research Ethics Committee (Tasmania) Network, ethics reference number H0017128. Officials of Oromia Regional State Health Bureau, South-West Shewa Zone Health Office, and Health Offices of the selected districts approved the data collection. Informed verbal consent was obtained from all study participants prior to data being collected.

## Results

### Participant characteristics

A total of 1303 people with LBP participated in this study, of whom 573 (44%) were females. Participants’ age ranged from 18–97 years, with a median (interquartile range [IQR] age of 38 years (30–50 years). Table 1 presents sociodemographic characteristics of the study participants.

**Table 1:** Sociodemographic characteristics of the study participants (n = 1303).

Characteristics	Number	%
**Gender**		
Male	730	56
Female	573	44
**Age (in years)**^♣^	38 (30–50)	-
**Ethnicity**		
Oromo	959	73.6
Amhara	214	16.4
Gurage	123	9.4
Others	7	0.5
**Educational level**		
No formal education	231	17.7
Elementary (grade 1–8)	544	41.7
Secondary (grade 9–12)	228	17.5
Technical/Vocational Certificate	74	5.7
Diploma	132	10.1
First degree or higher	94	7.2
**Residence**		
Urban	440	33.8
Rural	863	66.2
**Marital status**		
Single/never married	202	15.5
Married	908	69.7
Cohabited^Φ^	28	2.1
Separated	29	2.2
Divorced	45	3.5
Widowed	91	7.0
**Living condition**		
Living with nuclear family	1134	87.0
Living with nonnuclear family	44	3.4
Living alone	125	9.6
Household family size^♣^	4 (3–6)	-

^**♣**^Median (interquartile range);

^Φ^couple not officially married but living together as a wife/husband

### Content validity

The content validity index of the items forming the instrument ranged between 0.80 and 1.00 with modified Kappa coefficient ranged between 0.79 and 1.00. This shows that 80% to 100% of the validators valued the items as either “quite relevant” or “highly relevant” showing that the instrument is content valid.

### Factorial validity

All items of the instrument designed to investigate determinants of health care utilisation for LBP were subjected to PCA. Prior to performing PCA, the suitability of data for factor analysis was assessed. Inspection of the correlation matrix showed the presence of many coefficients above 0.3. The KMO measures of sampling adequacy value was 0.81, exceeding the recommended value of 0.6 [47] and Bartlett’s test of sphericity [48] reached statistical significance, supporting the factorability of the correlation matrix.

PCA with Varimax rotation demonstrated the presence of seven components with Eigenvalues exceeding one, explaining 20%, 11%, 9.1%, 7%, 6.5%, 5.1% and 3.9% of the variance, respectively. An inspection of the Scree plot also revealed a clear break after the 7^th^ component. This was further investigated using parallel analysis, which showed that only six components with Eigenvalues exceeding the corresponding criterion values for a randomly generated data matrix of the same size (Table 2). Using the results of parallel analysis, it was decided to retain six component solutions, which explained a total of 58.7% of the variance. Table 3 presents the final component loadings for the retained factors. Thus, the final PCA with Varimax rotation regrouped the initially developed items under the five broad domains into six specific components. Some terms of the original five different components were also loaded together. For example, two items, namely ‘health status in the past year’ and ‘current health status’, which were initially developed under the component of ‘general health related factors’ were loaded together with ‘pain related factors’. Accordingly, the new scale was renamed as ‘pain interrelated factors’.

**Table 2:** Comparison of Eigenvalues from PCA and criterion values from parallel analysis.

Component number	Actual Eigenvalue from PCA	Criterion value from parallel analysis	Decision
1	5.391	1.2734	Accept
2	2.975	1.2339	Accept
3	2.454	1.2058	Accept
4	1.890	1.1817	Accept
5	1.747	1.1578	Accept
6	1.382	1.1384	Accept
7	1.065	1.1177	Reject

PCA: Principal component analysis

**Table 3:** Results of PCA with Varimax rotation for the measurement instrument.

Scales/Items	Factors and item loadings (Varimax Rotation)					
	1	2	3	4	5	6
**Pain-interrelated factors**						
Intensity of the pain	**.788**	.050	-.065	.166	.044	.073
Health status in the past year	**.714**	.101	.067	.076	-.029	-.174
Pain interference with social activities	**.712**	.214	.226	.129	.093	-.227
Current health status	**.694**	.164	.144	.180	.040	.118
Pain interference with daily activities	**.693**	.214	.234	.156	.132	-.250
Duration of the pain	**.626**	-.225	-.033	.070	-.057	-.266
**Beliefs about LBP**						
LBP is not curable	.146	**.875**	.120	-.056	.045	.006
There is no real treatment for LBP	.092	**.755**	.099	-.105	-.011	-.052
LBP makes everything in life worse	-.044	**-.748**	-.043	.012	-.046	-.001
Health care providers cannot do anything for LBP	.129	**.716**	.113	-.047	.029	.072
LBP eventually stops you from working	.020	**-.644**	.046	-.117	-.012	.083
**Insomnia/sleeping problem**						
Difficulty of falling asleep at night	.142	.064	**.786**	.213	.077	-.023
Sleepiness during the day	.051	.097	**.779**	.120	.032	.018
Waking up too early and not getting back to sleep	.026	.038	**.725**	.049	.013	-.124
Waking up repeatedly during the night	.151	.114	**.570**	.282	.073	-.052
**Depressive symptoms**						
Feeling of hopelessness	.199	-.075	.120	**.781**	-.005	.175
Feeling depression	.065	.005	.138	**.739**	-.023	-.095
Feeling of worthlessness	.178	-.095	.231	**.610**	.079	-.092
Feeling of helplessness	.320	.075	.202	**.534**	.112	.049
**Health behaviour/lifestyle habits**						
Khat chewing	.061	.070	.089	.026	**.870**	-.115
Cigarette smoking	-.029	.057	-.006	.038	**.864**	-.051
Alcohol consumption	.114	-.029	.107	.042	**.670**	.347
**LBP associated sequelae**						
Additional spinal pain in another site(s)	-.144	.008	-.131	.130	.004	**.721**
Pain spread down the leg(s)	-.068	-.031	.043	-.243	.026	**.715**
Days off work due to the pain	-.294	-.048	-.156	.134	.011	**.684**

LBP: low back pain

### Internal consistency and test-retest reliability

Internal consistency reliability, which was measured by Cronbach’s *alpha* ranged from 0.65 for the scale ‘LBP associated sequelae’ (i.e. the only scale with an alpha less than 0.70) to 0.82 for the scale ‘beliefs about LBP’ (Table 4).

**Table 4:** Mean scores, standard deviations and internal consistency reliability (measured by Cronbach’s *alpha)* for the factorially derived domains/scales.

Domains/scales	Number of items	Mean (SD)	Cronbach’s *alpha*
Pain interrelated factors	6	19.69 (5.64)	0.81
Beliefs about LBP	5	17.25 (6.89)	0.82
Insomnia/sleeping problem	4	8.87 (2.43)	0.75
Depressive symptoms	4	8.30 (2.57)	0.72
Health behaviour/lifestyle habits	3	7.96 (1.58)	0.70
LBP associated sequelae	3	5.31 (0.94)	0.65

LBP: low back pain; SD: standard deviation

The results for the test-retest reliability showed that ICC (95% CI) and Cohen Kappa statistic (95% CI) varied from 0.60 (0.23–0.98) to 0.95 (0.81–1.00) and 0.72 (0.49–0.94) to 0.93 (0.85–1.00), respectively. Only in three items that ICC less than 0.70 while Kappa coefficient was greater than 0.70 in all items (Table 5).

**Table 5:** Test-retest reliability measured as ICC (95% CI) and Kappa (95% CI).

Scales/items	Test-retest reliability	
	ICC (95% CI)	Kappa (95% CI)
**Pain interrelated factors**		
Intensity of the pain	0.69 (0.38–0.94)	0.79 (0.66–0.92)
Health status in the past year	0.89 (0.68–0.99)	0.84 (0.69–1.00)
Pain interference with social activities	0.80 (0.50–0.98)	0.84 (0.74–0.93)
Current health status	0.91 (0.74–0.99)	0.93 (0.85–1.00)
Pain interference with daily activities	0.81 (0.53–0.98)	0.83 (0.73–0.94)
Duration of the pain	0.95 (0.81–1.00)	0.89 (0.67–1.00)
**Beliefs about LBP**		
LBP is not curable	0.80 (0.53–0.97)	0.85 (0.71–0.99)
There is no real treatment for LBP	0.78 (0.50–0.97)	0.85 (0.72–0.98)
LBP makes everything in life worse	0.71 (0.39–0.96)	0.80 (0.64–0.96)
Health care providers cannot do anything for LBP	0.85 (0.63–0.98)	0.89 (0.82–0.97)
LBP eventually stops you from working	0.63 (0.28–0.96)	0.73 (0.46–1.00)
**Insomnia/sleeping problem**		
Difficulty of falling asleep at night	0.76 (0.44–0.98)	0.83 (0.72–0.93)
Sleepiness during the day	0.76 (0.44–0.98)	0.82 (0.68–0.95)
Waking up too early and not getting back to sleep	0.71 (0.37–0.97)	0.78 (0.64–0.93)
Waking up repeatedly during the night	0.73 (0.39–0.98)	0.76 (0.61–0.91)
**Depressive symptoms**		
Feeling of hopelessness	0.83 (0.56–0.99)	0.81 (0.71–0.92)
Feeling depression	0.60 (0.23–0.98)	0.72 (0.49–0.94)
Feeling of worthlessness	0.76 (0.41–0.99)	0.79 (0.62–0.95)
Feeling of helplessness	0.83 (0.56–0.99)	0.84 (0.72–0.95)
**Health behaviour/lifestyle habits**		
Khat chewing	0.87 (0.52–1.00)	0.82 (0.59–1.00)
Cigarette smoking	0.95 (0.80–1.00)	0.88 (0.71–1.00)
Alcohol consumption	0.88 (0.56–1.00)	0.87 (0.70–1.00)
**LBP associated sequelae**		
Additional spinal pain in another site(s)	0.88 (0.55–1.00)	0.87 (0.70–1.00)
Pain spread down the leg(s)	0.94 (0.74–1.00)	0.79 (0.38–1.00)
Days off work due to the pain	0.89 (0.58-.00)	0.80 (0.54–1.00)

ICC: intraclass correlation coefficient

## Discussion

A comprehensive review of the literature demonstrated that health care utilisation for LBP is dependent of multiple factors [24], which can be broadly classified into sociodemographic, health behaviour/lifestyle habits, beliefs about LBP, pain and general health related factors, and factors related to accessibility to health services. Studies have so far attempted to characterise the impact of these factors on health care utilisation for optimal management of LBP in high-income countries [25, 27, 49, 50]. Epidemiological data to demonstrate factors influencing health care utilisation for LBP in the context of low-income countries are lacking [24]. In addition, the findings of previous studies reporting factors influencing health care utilisation for LBP lack consistency, particularly relating to sociodemographic factors [24, 25]. For example, previous studies in Japan [49] and Israel [51] indicated a statistically significant association between age and health care utilisation for LBP. Regardless of the difference in the statistical methods used to analyse the association between age and health care utilisation for LBP, both studies found similar results. The Japanese study found that the odds of medical care utilisation was 1.72, 95% CI: 1.04–2.84 and 2.47, 95% CI: 1.39–4.40 higher among the population aged 40–59 years and >60 years, respectively when compared with individuals younger than 40 years. Similarly, the Israeli study indicated that the prevalence rate of health care utilisation was higher in individuals 45–59 and >60 years (χ^2^ = 8.3, p< 0.041). In contrast, Mannion et al [52] found that there was no statistically significant association between age and health care utilisation for LBP. The findings reported the influence of gender on health care utilisation for LBP also showed inconsistency across studies. While in some studies [52–54] a significantly higher prevalence of health care utilisation was observed among females than males, a study undertaken by Ono et al [49] indicated that the influence of gender on health care utilisation was statistically not significant.

From the broad categories of factors influencing health care utilisation for LBP, matching results were observed in the literature only in relation to pain related factors. In particular, several studies consistently demonstrated that higher intensity of pain was associated with a higher prevalence rate of health care utilisation [25, 49, 50, 52]. Likewise, longer duration of pain was shown to be associated with increased rate of health care utilisation [25, 55, 56]. Furthermore, previous research has demonstrated that the prevalence rate of heath care utilisation for LBP varies between geographic regions of the world and within geographic areas of a country accounting for several factors described elsewhere [24]. For example, the fundamental differences between high and low-income countries, impact differently on LBP and associated health care utilisation [57]. This suggests that the development and validation of a measurement instrument for later investigation of determinants of health care utilisation for LBP in low-income countries may be worthwhile.

In research, clinical practice and health assessment, measurement instruments play a key role in deriving the required data [58, 59]. However, it is worth noting that the trustworthiness of the results obtained by measurement instruments comes from the reliability and validity of the instruments [60]. For this reason, these two fundamental concepts (reliability and validity) underpin the development of measurement instruments, from item generation to psychometric analyses [61, 62]. Rattray and Jones [61] noted that “when interpreting results from questionnaires, the development process should be defined in sufficient detail and with sufficient rigour to enable a practitioner to make an informed decision about whether to implement findings”. A psychometrically sound measurement instrument derives reliable and valid data that can be used to pursue generalisable truths, upon which practices and policy decisions can be formulated [63–65]. In light of this, it has been argued in the literature that the design and development of a measurement instrument need to be supported by a logical, systematic and structural approach [61]. The attainment of this argument, in turn seeks the strategies to demonstrate the reliability and validity of newly developed measurement instruments. Accordingly, this study was designed to develop and validate a theoretically anchored measurement instrument to investigate determinants of health care utilisation for LBP within the context of Ethiopia.

In a measurement instrument validation process, content validity assessment is an early step. As per the existing guidelines [39, 66] for content validity index of a measurement instrument, the newly developed instrument in this study demonstrated evidence of strong content validity.

PCA followed by parallel analysis produced six factor solutions explaining 58.7% of the variance. There is evidence that self-reported measures need to have internal consistency reliability of ≥ 0.70 when measured in Cronbach’s *alpha* [67]. To ascertain this notion, internal consistency reliability of the instrument was evaluated in this study, and the results showed that (except in one scale with Cronbach’s *alpha* of 0.65), in all the factorially derived scales, Cronbach’s *alpha* > 0.70 was observed. This provides evidence of a good level of internal consistency reliability of the instrument.

The literature recommends that the ICC is used to assess test-retest reliability of each item, with the instrument for clinical studies needing to be greater than 0.70 [68]. In accordance with this concept, (except in three items) the estimated ICC for test-retest reliability was greater than 0.70. Considering the possibility of agreement by chance, the test-retest reliability of the instrument was examined further by calculating Cohen Kappa coefficient, which was greater than 0.70 in all items of the instrument. Thus, the good to excellent ICC and Cohen Kappa coefficients indicate that the instrument is reliable to measure the corresponding scales constantly over time. This acceptable level of reliability and temporal stability of the instrument could be attributed to clarity, simplicity and specificity of the questions as suggested in research into questionnaire design [69]. This finding is also in accord with the evidence demonstrating that the process of developing and validating a measurement instrument primarily focussed on minimising error in the measurement process [44, 46].

This study has three specific strengths. Firstly, the study was conducted following a comprehensive review of the literature on health care utilisation for LBP and health care theories and models. This justified the study to using a theoretical framework derived from a combination of theories and models to identify domains and to develop and validate the instrument. Secondly, a multi-stage random sampling of socio-economically diverse population from both urban and rural residents ensures future generalisability of the instrument. Thirdly, the study demonstrated evidence of both content and factorial validity, internal consistency and temporal stability, which can be used by future searchers, particularly in Ethiopia, and other countries, with similar health care systems.

However, there are a few limitations to this study, including the small sample size used to evaluate temporal stability of the instrument. In addition, the applicability of this instrument for use in other population with different health care systems in other low-income countries is uncertain. Future studies about utilisation of other health care systems could be warranted.

## Conclusions

This study demonstrated that the newly developed measurement instrument has overall good level of psychometric properties measured as content and factorial validity, internal consistency reliability and temporal stability. The instrument is robust enough to investigate determinants of health care utilisation for LBP in Ethiopia. Investigating determinants of health care utilisation using this instrument may provide comprehensive information that will assist the development of appropriate strategies to improve health care utilisation behaviours of people with LBP. Such strategies may include LBP-focussed outreach programs consisting of community-based education enabling the individuals to receive appropriate and timely care. These programs can be implemented with the limited available resources by integrating into the already existing innovative community-based health program, called health extension packages. This would ultimately reduce the long-term impact of LBP on individuals and the society at large.

## Supporting information

S1 SPSS Dataset. (SAV)

S1 Text English version of the developed and validated measurement instrument. (DOCX)

S2 Text Translated (Oromo language) version of the developed and validated measurement instrument. (DOCX)
