# Supplementary material for: The development and validation of a measurement instrument to investigate determinants of health care utilisation for low back pain in Ethiopia
Source: PLoS One. 2020 Jan 16;15(1):e0227801. doi: 10.1371/journal.pone.0227801 (PMC6964895; doi:10.1371/journal.pone.0227801)
Supplement: S1 Text — (DOCX) [file pone.0227801.s002.docx]

**English version of the developed and validated measurement instrument to investigate determinants of health care utilisation for low back pain in Ethiopia**

1. **Socio-demographic data**
2. Gender  Male  Female
3. Age (in years) __________
4. Ethnicity __________
5. The highest level of education you have completed

No formal education  Technical/Vocational Certificate

Elementary (grade 1-8)  Diploma

Secondary (grade 9-12)  First degree or higher

1. Where do you live?  Urban  Rural
2. Current marital status

Single/never married  Cohabited  Divorced

Married  Separated  Widowed

1. With whom do you live?

Living with nuclear family  Living with nonnuclear family  Living alone

1. How many family members live in your household? _______
2. What is your current occupation, that is, what kind of work do you mainly do? _______
3. **Pain interrelated characteristics**
4. In general, how would you rate your LBP on average? ^*^

| 1 | 2 | 3 | 4 | 5 | 6 | 7 | 8 | 9 | 10 |  |
| --- | --- | --- | --- | --- | --- | --- | --- | --- | --- | --- |
|  | | | | | | | | | | |

No pain

Worst imaginable pain

1. How would you rate your health status in the past year?

Excellent  Very good  Good  Fair  Poor

1. How much did LBP interfere with your ability to participate in social activities? ^*^

Not at all  A little bit  Somewhat  Quite a bit  Very much

1. In general, how would you rate your current health status on average?

Excellent  Very good  Good  Fair  Poor

1. How much did LBP interfere with your day-to-day activities? ^*^

Not at all  A little bit  Somewhat  Quite a bit  Very much

1. How long has LBP been an ongoing problem for you? ^*^

Less than one month  Greater than 3 months, but less than one year

1–3 months  1–5 years  More than 5 years

1. **Beliefs about LBP**

To what extent do you agree with the following statements?

1. LBP is not curable

Strongly agree  Agree  Neutral  Disagree  Strongly disagree

1. There is no real treatment for LBP^‡^

Strongly agree  Agree  Neutral  Disagree  Strongly disagree

1. LBP makes everything in life worse^‡^

Strongly agree  Agree  Neutral  Disagree  Strongly disagree

1. Health care providers cannot do anything for LBP^‡^

Strongly agree  Agree  Neutral  Disagree  Strongly disagree

1. LBP eventually stops you from working^‡^

Strongly agree  Agree  Neutral  Disagree  Strongly disagree

1. **Insomnia/sleeping problem**

Please, tick the best option that describes your sleeping in the past year

1. Difficulty falling asleep at night

Never  Seldom  Sometimes  Several times

1. Sleepiness during the day

Never  Seldom  Sometimes  Several times

1. Waking up too early and not getting back to sleep

Never  Seldom  Sometimes  Several times

1. Waking up repeatedly during the night

Never  Seldom  Sometimes  Several times

1. **Depressive symptoms**

Please, tick the best option that describes how you have been feeling in the past year^*^

1. Hopeless  Never  Seldom  Sometimes  Several times
2. Depressed  Never  Seldom  Sometimes  Several times
3. Worthless  Never  Seldom  Sometimes  Several times
4. Helpless  Never  Seldom  Sometimes  Several times
5. **Health behaviour/lifestyle habits**
6. Khat chewing status

Chewing  Used to chew, but have now quit  Never chewed

- 1. Frequency of chewing khat  Occasionally  Often  Always

1. Cigarette smoking status

Current smoker  Used to smoke, but have now quit  Never smoked

- 1. For how long have you been smoking? _________ years or _______ months
  2. Number of cigarettes smoked per day _________

1. Alcohol consumption status  Drinking  Used to drink, but have now quit

Never drunk

- 1. Frequency of alcohol consumption

On regular bases (regular drinker)  Occasionally (social drinker)

1. **LBP associated sequelae**
2. Do have you have another spinal pain in other site(s)?

Yes  No

- 1. If yes, at which body part(s)?

Upper back  Neck  Shoulder  Elbow  Hand wrist  Knee  Ankle  Others specify _________

1. Has the pain (LBP) spread down your leg/s? ^*^  Yes  No
2. In the past one year, have you ever been off work due to your LBP?  Yes  No
   1. If yes, for how long have you been off work? ________ days or ______ weeks
3. **Health care utilisation data**
4. Have you ever sought consultation or health care from any health care provider for your LBP?  Yes  No

If your answer is ‘No’ to the above question, please, go to question number 9.

1. Where have you sought consultation or health care for your LBP?

Health post  General hospital

Health centre  Comprehensive specialised hospital

Primary hospital  Private clinic

1. Have you ever had a low back operation?  Yes, one operation  Yes, more than one operation  Never
2. In the past one year, have you sought consultation or healthcare from any health care provider for your current LBP?  Yes  No

If your answer is ‘No’ to the above question, please, go to question number 9.

1. Where have you sought consultation or healthcare for your LBP?

Health post  General hospital

Health centre  Comprehensive specialised hospital

Primary hospital  Private clinic

1. In the past one year, how many times have you sought consultation or healthcare from the health care provider for your current LBP? ________
2. What type of treatment was prescribed for your LBP?

Injection medicine  Surgery  Back support  Bed rest

Exercise  Massage  Others, specify _____________

1. In the past one year, have you been hospitalised due to your LBP?  Yes  No
   1. If ‘Yes’, for how many days have you been stayed in the hospital? __________
2. In the past one year, have you used any traditional medicine for your current LBP?  Yes  No
   1. If ‘Yes’, please, list the traditional medicines that you have used. ________________

Note:

*-Items taken/adapted from:

Deyo RA, Dworkin SF, Amtmann D, Andersson G, Borenstein D, Carragee E, et al. Report of the NIH task force on research standards for chronic low back pain. Physical Therapy. 2015;95(2):e1-e18. doi: 10.2522/ptj.2015.95.2.e1.

‡-Items adapted from:

Bostick G, Schopflocher D, Gross D. Validity evidence for the back beliefs questionnaire in the general population. European Journal of Pain. 2013;17(7):1074-81. doi: 10.1002/j.1532-2149.2012.00275.x.
