# Supplementary material for: The development and validation of a measurement instrument to investigate determinants of health care utilisation for low back pain in Ethiopia
Source: PLoS One. 2020 Jan 16;15(1):e0227801. doi: 10.1371/journal.pone.0227801 (PMC6964895; doi:10.1371/journal.pone.0227801)
Supplement: S2 Text — (DOCX) [file pone.0227801.s003.docx]

**Etoophiyaa keessatti wantota sababa dhukkuba dugdaatiif wal’aansa fayyaa fayyadamuuu irratti dhiibbaa geessisuu danda’an qorachuuf gaafannoo karaa saayinsaawaa ta’een qophaa’ee mirkanaa’e**

1. **Gaafiiwwan hawaasummaa ilaallatan**
2. Koorniyaa  Dhiira  Dhalaa
3. Umrii (waggaan) __________
4. Qomoo __________
5. Sadarkaa barumsaa

Barumsa idilee kan hin baranne

Sadarkaa tokkoffaa (kutaa 1-8)

Sadrkaa 2ffaa (kutaa 9-12)

Tekinikaa fi ogummaa irraa kan eebbifame/eebbifamte

Dippiloomaa

Digrii jalqabaa ykn isaa ol

1. Bakka jireenyaa  Magaalaa  Baadiyyaa
2. Haala fuudhaa fi heerumaa

Gonkumaa kan hin fuune/hin heerumne

Kan fuudhee/heerumtee waliin jiraachaa jiran

Osoo wal hin fuudhiin kan waliin jiraachaa jiran

Fuudhee/heerumtee kan addaan bahanii garuu seeraan wal hin hiikne

Fuudhee/heerumtee kan wal-hiikan

Kan abbaan manaa irraa du’e/haati manaa jalaa duute

1. Eenyu wajjin jiraatta?

Maatii koo wajjin  Nama biraa kan maatii koo hin taane wajjin  Kophaa koo

1. Mana keessan keessa maatii meeqa taataniit jiraattu? _________
2. Yeroo ammaa kana irra caalaatti hojii maaalii irratti bobbaatee jirta? ___________
3. **Wantota dhukkuba dugdaatiin walqabatan**
4. Walumaagalatti, dhukkuba dugdaa kee kana qabxiilee armaan gadii irratti hundaa’uun akkamitti agarsiifta?

Dhukkubbii

kan hin qabne

Dhukkubbii

gar-malee/hamaa ta’e

| 1 | 2 | 3 | 4 | 5 | 6 | 7 | 8 | 9 | 10 |  |
| --- | --- | --- | --- | --- | --- | --- | --- | --- | --- | --- |
|  | | | | | | | | | | |

1. Fayyummaa kee waggaa darbee akkamitti ibsita?

Baay’ee baay’ee gaariidha  Baay’ee gaariidha  Gaariidha  Gahaadha  Yaraadha

1. Dhukkubni dugdaa dalagaa hawaasummmaa irratti hirmaannaa kee hagam gufachiise?

Gonkumaa na hin gufachiifne  Baay’ee xiqqoo na gufachiise

Hanga tokko na gufachiise  Xiqqoo ishee na gufachiise  Baay’ee na gufachiise

1. Walumaagalatti, fayyummaa kee yeroo ammaa kana akkamitti ibsita?

Baay’ee baay’ee gaariidha  Baay’ee gaariidha  Gaariidha  Gahaadha  Yaraadha

1. Dhukkubni dugdaa dalagaa idilee kee irraa hagam si gufachiise?

Gonkumaa na hin gufachiifne  Baay’ee xiqqoo na gufachiise

Hanga tokko na gufachiise  Xiqqoo ishee na gufachiise  Baay’ee na gufachiise

1. Dhukkubni dugdaaa erga si qabee hagam sirra ture?

Ji’a tokkoo gadi  Ji’a sadii ol, wagga tokkoo gadi

Ji’a tokkoo hanga ji’a sadii  Wagga tokkoo hanga shanii  Wagga shanii ol

1. **Dhukkuba dugdaa irratti amantaa jiru**

Himoota armaan gadii hagam sirriidha ykn sirrii miti jettee itti amanta?

1. Dhukkuba dugdaa irraa fayyuun hin danda’amu

Sirriidha jedhee baay’een amana  Sirriidha jedheen amana

Sirriidhas sirrii mitis jedhee hin amanu  Sirrii miti jedheen amana

Sirrii miti jedhee baay’een amana

1. Dhukkubni dugdaa wal’aansa hin qabu

Sirriidha jedhee baay’een amana  Sirriidha jedheen amana

Sirriidhas sirrii mitis jedhee hin amanu  Sirrii miti jedheen amana

Sirrii miti jedhee baay’een amana

1. Dhukkubni dugdaa waa’ee jireenyaa hunda mancaasa

Sirriidha jedhee baay’een amana  Sirriidha jedheen amana

Sirriidhas sirrii mitis jedhee hin amanu  Sirrii miti jedheen amana

Sirrii miti jedhee baay’een amana

1. Ogeeyyiin fayyaa dhukkuba dugdaatiif homaa gochuu hin danda’anu

Sirriidha jedhee baay’een amana  Sirriidha jedheen amana

Sirriidhas sirrii mitis jedhee hin amanu  Sirrii miti jedheen amana

Sirrii miti jedhee baay’een amana

1. Dhukkubni dugdaa deemee deemee walumaagalatti hojii nama dhorka

Sirriidha jedhee baay’een amana  Sirriidha jedheen amana

Sirriidhas sirrii mitis jedhee hin amanu  Sirrii miti jedheen amana

Sirrii miti jedhee baay’een amana

1. **Rakkoo hirribaa**

Waggaa darbe keessa, waa’ee hirriba kee ilaalchisee, filannoowwan kennaman keessaa isa sirriitti mudannoo kee ibsu filachuun agarsiisi

1. Halkan hirriba dhabuu

Gonkumaa na hin mudanne  Darbee darbee na mudataa ture

Yeroo tokko tokko na mudataa ture  Yeroo baay’ee na mudataa ture

1. Yeroo guyyaa hirribni si qabuu

Gonkumaa na hin mudanne  Darbee darbee na mudataa ture

Yeroo tokko tokko na mudataa ture  Yeroo baay’ee na mudataa ture

1. Barii barraaqa hirriba irraa dammaqxee hirribni deebi’ee si qabuu dhiisuu

Gonkumaa na hin mudanne  Darbee darbee na mudataa ture

Yeroo tokko tokko na mudataa ture  Yeroo baay’ee na mudataa ture

1. Halkan hirriba keessaa dadammaquu

Gonkumaa na hin mudanne  Darbee darbee na mudataa ture

Yeroo tokko tokko na mudataa ture  Yeroo baay’ee na mudataa ture

1. **Mallattoowwan mukaa’uu**

Tokkoon tokkoo qabxiilee armaan gadii irratti, waggaa darbe keessa hagam akka sitti dhaga’amaa ture filannoowwan kennaman keessaa isa sirriitti ibsu filachuun agarsiisi

1. Abdi-dhabeessa/abdi-dhabeettii

Gonkumaa natti hin dhaga’amne  Darbee darbee natti dhaga’amaa ture

Yeroo tokko tokko natti dhaga’amaa ture  Yeroo baay’ee natti dhaga’amaa ture

1. Mukaa’uu

Gonkumaa natti hin dhaga’amne  Darbee darbee natti dhaga’amaa ture

Yeroo tokko tokko natti dhaga’amaa ture  Yeroo baay’ee natti dhaga’amaa ture

1. Gati-dhabeessa/Gati-dhabeettii

Gonkumaa natti hin dhaga’amne  Darbee darbee natti dhaga’amaa ture

Yeroo tokko tokko natti dhaga’amaa ture  Yeroo baay’ee natti dhaga’amaa ture

1. Gargaarsa-dhabeessa/Gargaarsa-dhabeettii

Gonkumaa natti hin dhaga’amne  Darbee darbee natti dhaga’amaa ture

Yeroo tokko tokko natti dhaga’amaa ture  Yeroo baay’ee natti dhaga’amaa ture

1. **Araada fayyaan walqabatan/haala jireenyaa**
2. Jimaa qama’uu

Nan qama’a  Qama’aan ture, amma garuu dhiiseen jira

Gonkumaa qama’ee hin beeku

- 1. Ni qamaata yoo ta’e, Jimaa yeroo hammamiitti qamaata?

Darbee darbee  Yeroo baay’ee  Yeroo mara

1. Tamboo xuuxuu

Nan xuuxa  Xuuxaan ture, amma garuu dhiiseen jira

Gonkumaa xuuxee hin beeku

- 1. Ni xuuxxa yoo ta’e, erga xuuxuu jalqabdee waggaa/ji’a meeqa ta’e?

wagga _______ ykn ji’a _______

- 1. Ni xuuxxa yoo ta’e, guyyaatti sijaaraa meeqa xuuxxa? __________

1. Dhugaatii alkoolii

Nan dhuga  Dhugaan ture, amma garuu dhiiseen jira

Gonkumaa dhugee hin beeku

- 1. Ni dhugda yoo ta’e, yeroo hammamiitti dhugda?

Yeroo mara  Darbee darbee

1. **Miidhaa dhukkuba dugdaatiin walqabatan**
2. Dhukkuba dugugguruu bakka biraa irraa qabdaa?  Eeyyee  Lakki
   1. Ni qabda yoo ta’e, bakka kam irraa?

Dugda gara ol-aanu  Morma  Gateettii  Ciqilee  Burrisa harkaa

Jilba  Burrisa miilaa (koroonyoo)  Bakka biroo, caqasi _________

1. Dhukkubbiin dugdaa kee kun gara miilaatti gadi faffaaca’eeraa?  Eeyyee  Lakki
2. Wagggaa darbe keessa, sababa dhukkuba dugdaatiin hojii irraa haftee beektaa?

Eeyyee  Lakki

- 1. Gaaffii armaan olii ‘Eeyyee’ jechuun deebifte yoo ta’e, yeroo hammamiif hojii irraa hafte? Guyyaa ______ ykn torban ______

1. **Gaafiiwwan wal’aansa fayyaa fayyadamuu ilaallatan**
2. Dhukkuba gugdaa keetiif takkaa gorsa yookiin wal’aansa ogeessa fayyaa argattee beektaa?

Eeyyee  Lakki

Gaaffii armaan olii ‘Lakki’ jechuun deebifte yoo ta’e, gara gaaffii lakkoofsa 9 tti ce’i.

1. Gorsa yookiin wal’aansa ogeessa fayyaa eessaa argatte?

Kellaa fayyaa  Hospitaala waliigalaa

Buufata fayyaa  Hospitaala tajaajila addaa

Hospitaala jalqabaa  Kilinika dhuunfaa

1. Dugda kee irra waldhaansi baqaqsanii yaaluu takkaa siif godhamee beekaa?

Eeyyee  Lakki

1. Waggaa darbe keessa, dhukkuba dugdaa kee isa ammaa kanaaf gorsa yookiin wal’aansa ogeessa fayyaa argattee beektaa?  Eeyyee  Lakki

Gaaffii armaan olii ‘Lakki’ jechuun deebifte yoo ta’e, gara gaaffii lakkoofsa 9 tti ce’i.

1. Gorsa yookiin waldhaansa ogeessa fayyaa eessaa argatte?

Kellaa fayyaa  Hospitaala waliigalaa

Buufata fayyaa  Hospitaala tajaajila addaa

Hospitaala jalqabaa  Kilinika dhuunfaa

1. Waggaa darbe keessa, gorsa yookiin waldhaansa ogeessa fayyaa al meeqa argatte? ____
2. Dhukkuba dugda keetiif qoricha/waldhaansa akaakuu kamiitu siif ajajame/kenname?

Qoricha lilmeen kennamu  Sochii qaamaa

Waldhaansa baqaqsanii yaaluu  Sukkuummii/dhidhiibbaa

Utubbii dugdaa  Kan biroo, caqasi _____

Boqonnaa siree irraa

1. Waggaa darbe keessa, sababa dhukkuba dugdaa keetiif hospitaalaa galtee ciiftee beektaa?  Eeyyee  Lakki
   1. Gaaffii armaan olii ‘Eeyyee” jechuun deebifte yoo ta’e, guyyaa meeqaaf hospitaala ciifte? __________
2. Waggaa darbe keessa, dhukkuba dugdaa kee isa ammaa kanaaf wal’aansa ykn qoricha aadaa fayyadamtee beektaa?  Eeyyee  Lakki
   1. Gaaffii armaan olii ‘Eeyyee’ jechuun deebifte yoo ta’e, wal’aansa ykn qoricha aadaa fayyadamte eeri ___________________
